# Extensive locally invasive nasopharyngeal carcinoma involving 10 cranial nerves palsies: an interesting case report

**DOI:** 10.3389/fonc.2025.1545838

**Published:** 2025-07-31

**Authors:** Abdulrazaq Albilali, Naif H. Alotaibi, Abdulaziz Alfadley, Khalid M. Alqarni, Abdullah Alhajlah

**Affiliations:** ^1^ Neurology Unit, Department of Medicine, College of Medicine, King Saud University, King Saud University Medical City, Riyadh, Saudi Arabia; ^2^ Department of Otolaryngology-Head & Neck, King Faisal Specialist Hospital and Research Centre, College of medicine, Alfaisal University, Riyadh, Saudi Arabia; ^3^ Department of Otorhinolaryngology, Imam Abdulrahman Bin Faisal University Hospital, Dammam, Saudi Arabia; ^4^ Department of Medicine and Surgery, College of Medicine, Imam Muhammad Ibn Saud Islamic University, Riyadh, Saudi Arabia

**Keywords:** nasopharyngeal carcinoma, cranial nerve palsies, chemoradiotherapy invasive nasopharyngeal squamous cell carcinoma, 10 cranial nerve palsies, chemoradiotherapy, case report

## Abstract

**Background and objectives:**

Cranial nerve palsies occur in approximately 20% of nasopharyngeal carcinoma (NPC) cases, often correlating with tumor location and cranial extension. This report describes a rare case involving ten unilateral cranial nerves.

**Methods:**

A 55-year-old female presented with right-sided cranial nerve palsies (II, III, IV, V, VI, VII, IX, X, XI, and XII). Imaging showed a locally invasive nasopharyngeal mass with anterior, posterior, and intracranial extension but without distant metastasis. Biopsy confirmed a poorly differentiated, non-keratinizing squamous cell carcinoma, staged as IV/A T4-N2-M0. Treatment involved concurrent chemoradiotherapy and multidisciplinary care.

**Results:**

Six months post-treatment, there was complete recovery in cranial nerves XI and XII and near-complete recovery in nerves III, IV, and VI. Cranial nerves II, V, VII, IX, and X showed no improvement at interim follow-up.

**Discussion:**

This case highlights an uncommon presentation of NPC with extensive cranial nerve involvement and no distant metastasis. Partial recovery of cranial nerve function following chemoradiotherapy emphasize the potential for neurological improvement in advanced NPC with comprehensive, multidisciplinary care.

## Introduction

Nasopharyngeal carcinoma (NPC) is a rare malignancy with a reported incidence of 1 in 100,000 and is two to three times more prevalent in males than in females (1). It commonly arises from the fossa of Rosenmüller. Owing to this anatomical location and the tumor’s ability to infiltrate, patients may exhibit a diverse array of symptoms, such as neck masses, nasal and visual disturbances, headaches, and cranial nerve (CN) impairments (2). The involvement of CN can occur in the first presentation of the disease; later in the disease progression, indicating a poor prognosis; or as an adverse effect resulting from radiation and chemotherapy treatments (3).

## Case presentation

**Figure 1:**
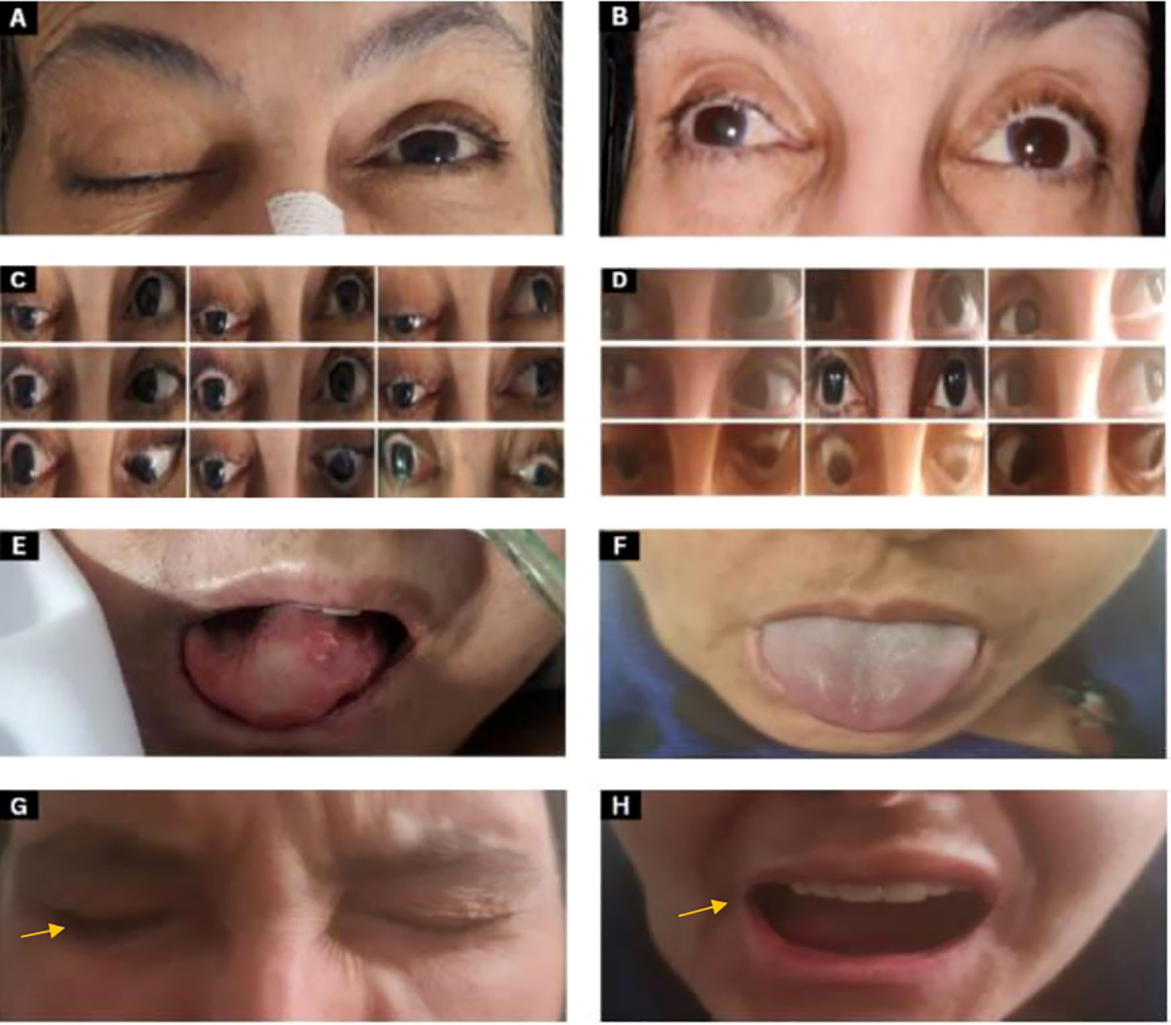
Clinical images pre and post-CRT **(A)** CN III examination shows complete ptosis of the right eye. **(B)** Anisocoria: the right eye pupil size is 5 mm while the left eye pupil size is 2 mm 6 months post-chemoradiation. **(C)** Two months after the onset of symptoms, extraocular movements show a frozen right eye (deficit of extraocular movements in all directions when asked to follow these arrows directions 
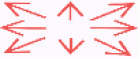
) with ptosis (eyes look towards the arrows). **(D)** Six months post-chemoradiation showed improvement of extraocular movements in all directions in the right eye and improved ptosis. **(E)** CN XII examination: tongue deviation to the right side with atrophy at the time of presentation. **(F)** The tongue shows improvement after 6 months post-chemoradiation. **(G, H)** CN VII examination shows right orbicularis oculi weakness during eye closure with incomplete burying of eyelashes and drooping of the right angle of the mouth as indicated by the arrow.

A 55-year-old female patient, previously healthy, was referred to us due to the rapid progression of a constellation of clinical symptoms that had occurred over 2 months. The sequence of symptoms was as follows: throbbing right facial pain for 2 days that was exacerbated by chewing, followed by right facial swelling, right eyelid ptosis, and right-side mouth droop for 1 week, as well as by progressive dysphonia, dysphagia affecting her oral intake, progressive blurriness of vision in the right eye, and right ear hyperacusis for 6 weeks. Negative family history for tumors. Neurological examination revealed the patient to be alert and oriented with intact language and memory. Multiple CN exams were abnormal (see **Table 1**, **Figure 1**). The rest of the neurological examination, including motor, sensory, coordination, and gait, was normal.

**Table 1:** Cranial Nerve Examination.

Cranial nerve	Clinical finding
I	N/A*
II	Right direct and Indirect pupil reflexes are impaired, blind to light and grade 1 papilledema
III	Right eye extraocular movement impairment in all directions with complete ptosis and dilated pupil measuring 8 mm
IV	Impaired downward inward movement of right eyeball
V	Pin-prick sensations in V1, V2, and V3 are reduced along with weakness of temporalis and masseter muscles and absent corneal reflex on the right side
VI	Impaired abduction in right eyeball
VII	Weakness in frontalis, orbicularis oculi, and orbicularis oris muscles, absent corneal reflex and hyperacusis on right side; impaired taste sensation in the anterior two-thirds of tongue
VIII	N/A**
IX	Absent gag reflex, depressed right palate and impaired taste sensation in posterior third of tongue
X	Absent gag reflex and hoarseness
XI	Weakness in shoulder shrug and head turn to the left
XII	Atrophy and tongue deviation to the right with weakness against contralateral resistance

*The olfactory function could not be assessed clinically due to the presence of a nasogastric tube.

**Weber and Rinne tests were refused by the patient due to hyperacusis.

EOM, extraocular movements; mm, millimetres.

**Figure 2:**
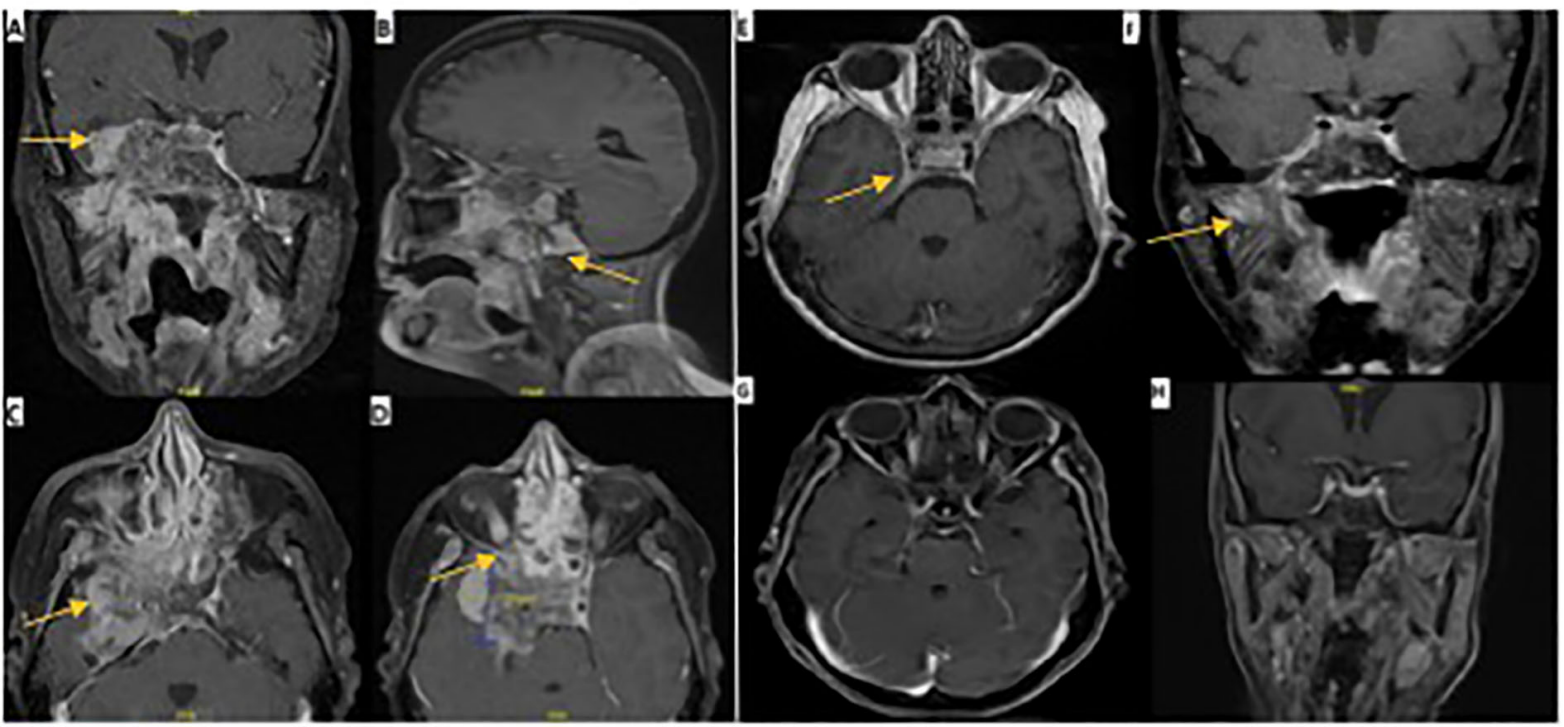
MRI images pre and post-CRT. Pre-ICT: **(A)** axial, coronal, and sagittal views of T1 with contrast MRI brain showing an aggressive mass invading the temporal lobe intracranially. **(B)** extension to the skull base. **(C)** extension to cavernous sinus. **(D)** extension to superior orbital fissure. One month post-ICT: **(E, F)** demonstrate axial and coronal views of T1 with contrast 4 months post-ICT with significant regression of tumor size and few remnants as indicated by the arrows. Eight months post-CRT: **(G, H)** demonstrate axial and coronal views of T1 with contrast with almost complete resolution of tumor.

Radiological assessment by MRI of the nasopharynx revealed a nasopharyngeal mass with extensive involvement primarily on the right side, extending into the parapharyngeal region and central skull base with the destruction of the clivus, occipital condyle, and right petrous apex. The bilateral sphenoid sinuses, pterygopalatine fossa, and pterygoid plates are infiltrated with extension into the right orbital apex. Intracranial extension was present, with invasion of the cavernous sinus and right Meckel’s cave, along with extension into the right temporal fossa, accompanied by vasogenic edema in the right temporal lobe (**Figure 2**).

A tissue biopsy was performed, and three specimens were collected. Histopathological examination revealed poorly differentiated, non-keratinizing squamous cell carcinoma. Immunohistochemistry was positive for P63 and CK5/6 and negative for CK-7, CK-20, and EBV. A CT of the chest, abdomen, and pelvis showed no distant metastasis, while a PET-CT scan showed multiple bilateral cervical lymph node uptake. Imaging and biopsy-based staging revealed a locally advanced nasopharyngeal squamous cell carcinoma without metastasis stage IV/A T4-N2-M0.

The case was discussed on the tumor board, and a decision for concurrent chemoradiotherapy (CRT) was made. The patient received three cycles of induction chemotherapy. The initial cycle included induction chemotherapy (ICT) with gemcitabine and cisplatin, followed by two subsequent cycles of gemcitabine and carboplatin. A cumulative radiation dose of 6996 centigray (cGy) dispersed across 33 fractions was prescribed which were tolerable with no reported toxicity. We contoured the CT image obtained for simulation in our radio-oncology department (after chemotherapy), then we fused it with MRI and PET scans pre-chemotherapy. Rather than contouring individual cranial nerves, which are not well visualized on imaging, we contoured anatomical structures based on their anatomical relationship with neural pathways. These include foramina and bony landmarks (4). The patient was concurrently undergoing maintenance therapy with weekly cisplatin. The regimen of CRT is detailed in (**Table 2**).

**Table 2:** Summery of induction chemotherapy and chemoradiotherapy.

Date	Chemoradiotherapy
Cycle #1 23/9/2023	Day 1: -Gemcitabine 800mg/m2 over 30 min (20% reduced from1000mg/m2) -Cisplatin 25mg/m2 over 60 min Day 8: -Gemcitabine 800mg/m2 over 30 min Cisplatin 25mg/m2 over 60 min
Cycle #2 22/11/2023	Day 1: -Gemcitabine 1000mg/m2 with 30% reduction + carboplatin AUC 4 -Gemcitabine 1000mg/m2 with 30% reduction Day 8: -Gemcitabine 1000mg/m2 with 30% reduction
Cycle #3 19/12/2023	Day 1: -Gemcitabine 1000mg/m2 with 30% reduction + carboplatin AUC 4 Day 8: -Gemcitabine 1000mg/m2 with 30% reduction
10/3/2024 - 28/4/2024	6 doses of weekly cisplatin 50mg/m2 with 30% dose reduction (week 4 was omitted due to neutropenia)
10/3/2024 - 29/4/2024	Radiotherapy prescribed 6996 cGy in 33 fractions, 212 cGy per fraction, 5 days a week for 6.5 weeks, to the head and neck with lymph nodes with Volumetric Modulated Arc Therapy (VMAT) technique. Some pauses were due to technical machine issues and patient was compensated with BID fractions at the last day of her treatment.

Follow-up at 6 months showed significant improvement in right eyelid ptosis. All extraocular movements of the right eye almost completely recovered, and there was complete recovery in shoulder shrugging and head turning. Oral examination revealed normal tongue power, without deviation or atrophy. These findings indicate near-complete recovery of CN III, IV, and VI and complete recovery of CN XI and XII (**Figure 1**). Although depression and anxiety scales were not used during the follow up visit, patient did confirm that she feels much better compared when she was seen first six months ago, as she mentioned during her last visit “I feel a lot better now”. Two follow-up MRI images were done. The first one was one month post-ICT which revealed significant tumor size regression with few remnants and the second was 8 months post-CRT with almost complete resolution of tumor (**Figure 2**).

## Discussion

We present a rare case of locally advanced NPC stage IV/A T4-N2-M0 with no distant metastasis involving 10 unilateral CN palsies (II, III, IV, V, VI, VII, IX, X, XI, and XII), indicative of extensive anterior and posterior skull base and intracranial involvement. To the best of our knowledge, this report is the first of a case of NPC involving 10 CN with recovery of 5 CN within 6 months post-chemoradiation.

Nasopharyngeal carcinoma most commonly affects CN VI (56.4%) and V2 (47.9%) and V3 (29.2%) branches of CN V, and it rarely affects CN II (8.6%) or VII (2.3%) (5). The CN deficits from NPC depends mainly on the anatomical extension patterns of the tumor. For example, an extension of the tumor into the orbital apex and cavernous sinus affects CN II, III, IV, V, and VI and leads to impaired vision, ophthalmoplegia, and facial sensory dysfunction (2). An extension to the cerebellopontine angle, the middle ear, or the parotid can involve CN VII (6). The presentation of CN VII palsy may differ depending on which nerve segment is affected during its anatomical course (7). Tumor extension into the jugular foramen affects CN IX, X, and XI, leading to dysphagia, vocal cord palsy, and weakness of the trapezius and sternocleidomastoid muscles (2). Involvement of the hypoglossal canal affects CN XII and presents with ipsilateral tongue deviation (8). One previous case reported involvement of six CN (III, V, VI, VII, IX, and XI) (7). Another study reported the involvement of nine unilateral CN (III, IV, V, VI, VII, VIII, IX, X, XII) as a Garcin’s Syndrome secondary to NPC with improvement post-CRT. However, the extent of clinical and radiological improvement was not detailed (9).

In our case, the involvement of CN II, III, IV, V, and VI was explained by the anterior tumor extension to the orbital apex and cavernous sinus as described by MRI. The patients’ optic neuropathy, ptosis, complete ophthalmoplegia, and hypoesthesia of the ophthalmic distribution of V1 on the right side are consistent with orbital apex syndrome that has been described in the literature (10). In addition, the involvement of the lower divisions of the right CN V in this case was represented by the lower facial pain and hypoesthesia across the distribution of V2 and V3 along with weakness of the masseter and temporalis muscles on the right side. These symptoms are indicative of tumor infiltration into the superior orbital fissure, foramen rotundum, and foramen ovale (5). The posterior extension of the tumor resulted in the involvement of CN VII, IX, X, XI, and XII by compressing the internal auditory meatus, jugular foramen, and hypoglossal canal (2, 8).

A multidisciplinary team should discuss the appropriate treatment approach for patients with advanced NPC (11). Concurrent chemoradiation is the cornerstone treatment protocol for patients diagnosed with advanced, non-metastatic NPC, whereas radiotherapy plays a major role in locoregional therapy (11). According to the European Society of Medical Oncology, Stages III and IV/A are commonly managed with CRT with cisplatin as the agent of choice. At such stages, induction chemotherapy with cisplatin and gemcitabine followed by CRT has been shown to result in better overall survival, recurrence-free survival (RFS), and distant RFS (12). Our patient was not a candidate for surgical resection due to the complex anatomy of this region. However, a multidisciplinary team, including various specialties, participates in the management of different systems. In addition, the patient received three cycles of induction chemotherapy followed by CRT as described in the case presentation.

The recovery of CN in advanced NPC post-CRT was evaluated, and it was concluded that the oculomotor, trochlear, and abducens nerves have the highest 5-year full recovery rate. In addition, involvement of the optic nerve, smoking, and more than 2 months of neuropathy are all negative predictors of recovery (5). Our patient exhibited a near-complete recovery of CN III, IV, and VI, and a complete recovery of CN XI and XII at the 6-month follow-up due to the significant response to the therapy and regression of the tumor extensions. Regarding psychological complications from NPC, it has been reported that up to a third of patients with NPC have anxiety and depression (13). When our patient was asked about her neurological and depressive symptoms post-CRT, she replied “I feel a lot better now”. To our knowledge, complete recovery, or near-complete recovery of a total of five CN in advanced NPC has not been described previously in the literature. In addition, our case report supports that the current recommended chemo-radiation regimen is capable of treating extensive NPCs with complete or near-complete recovery of some the impaired CN.

## Data Availability

The datasets presented in this article are not readily available because of ethical and privacy restrictions. Requests to access the datasets should be directed to the corresponding author/s.
